# Supplementary material for: Dissecting the role of PfAP2-G in malaria gametocytogenesis
Source: Nat Commun. 2020 Mar 20;11:1503. doi: 10.1038/s41467-020-15026-0 (PMC7083873; doi:10.1038/s41467-020-15026-0)
Supplement: Supplementary file 1 — Supplementary Information [file 41467_2020_15026_MOESM1_ESM.pdf]

Dissecting the role of PfAP2-G in malaria gametocytogenesis

Josling *et al*.

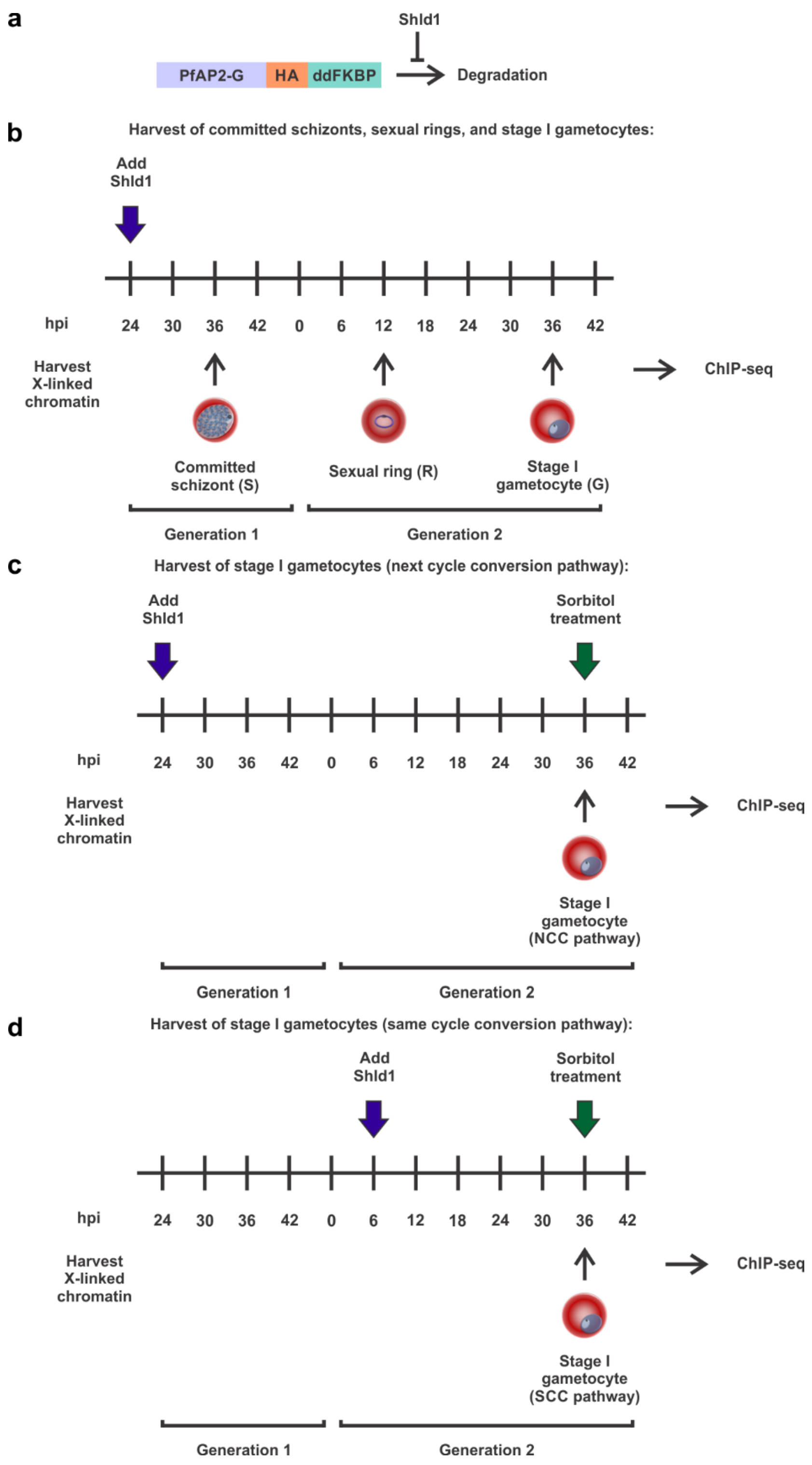

Supplementary Figure 1: Schematic showing the design of the ChIP-seq experiments using AP2-G-DD. The AP2-G-DD line[1] was used (**a**), as it allows for conditional stabilisation or degradation of PfAP2-G depending on the presence of Shld1. For the first set of experiments (**b**), Shld1 was added to trophozoites to induce commitment and chromatin was harvested at three stages: committed schizonts (S), sexual rings (R), and stage I gametocytes (G). For the second

set of experiments (**c** and **d**), Shld1 was added either at the trophozoite stage or the early ring stage to induce next-cycle conversion (NCC) and same-cycle conversion (SCC), respectively, as previously described[2]. In the following cycle, cultures were treated with sorbitol prior to crosslinking and isolating chromatin to selectively kill trophozoites and schizonts.

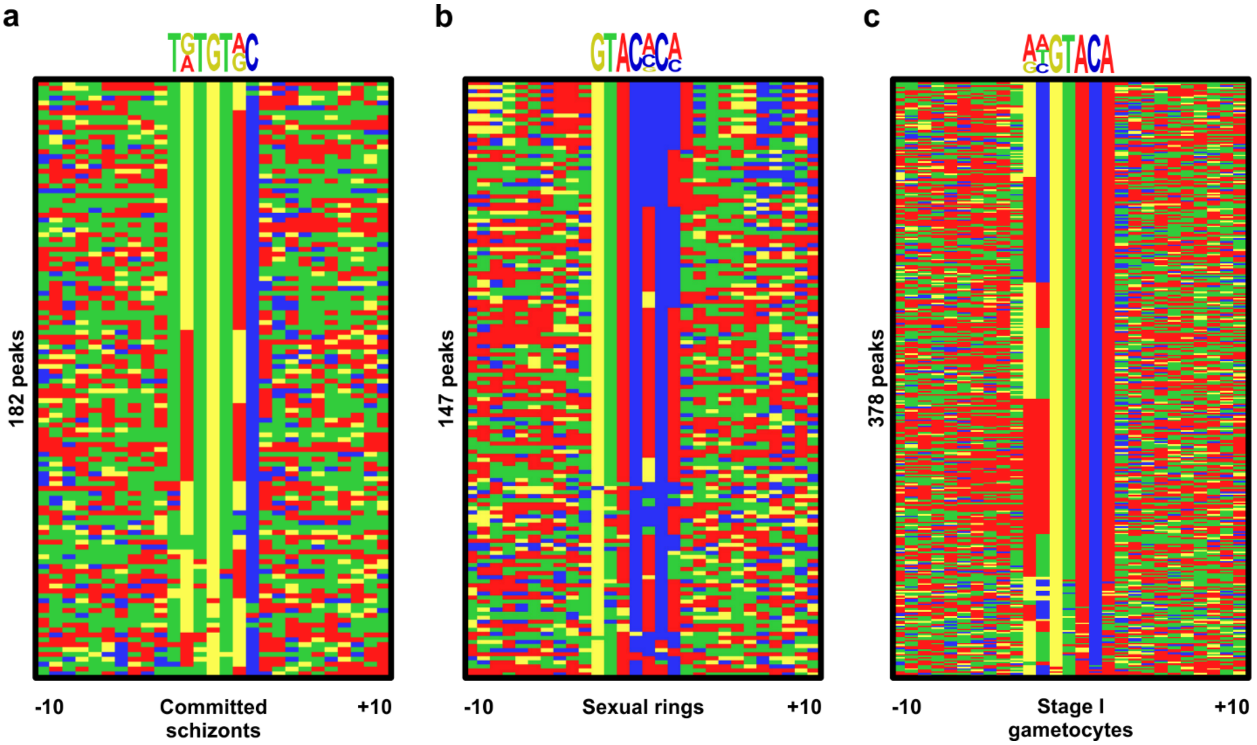

Supplementary Figure 2: DNA motif heatmaps of PfAP2-G ChIP-seq peaks and motifs. a, committed schizonts, **b**, sexual rings, and **c**, stage I gametocytes. Only peaks with a motif were used for this analysis.

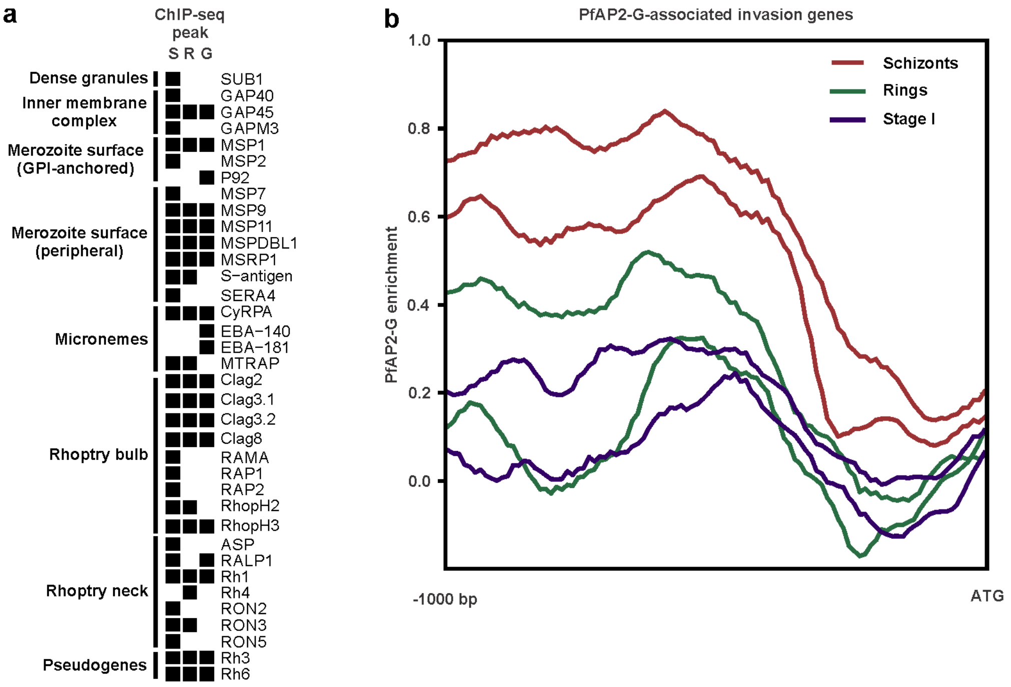

Supplementary Figure 3: PfAP2-G enrichment in invasion gene promoters. **a**, Invasion genes that are bound by PfAP2-G in one or more stage are listed on the left and are organized by compartment. The black squares to the right indicate whether or not the gene has a ChIP-seq peak upstream of it in each of the three stages tested. **b**, Plot showing the average enrichment of PfAP2-G across the 1000 bp upstream of the 36 invasion genes bound by PfAP2-G. Both biological replicates are shown for each stage, with enrichment in committed schizonts in red, sexual rings in green, and stage I gametocytes in purple.

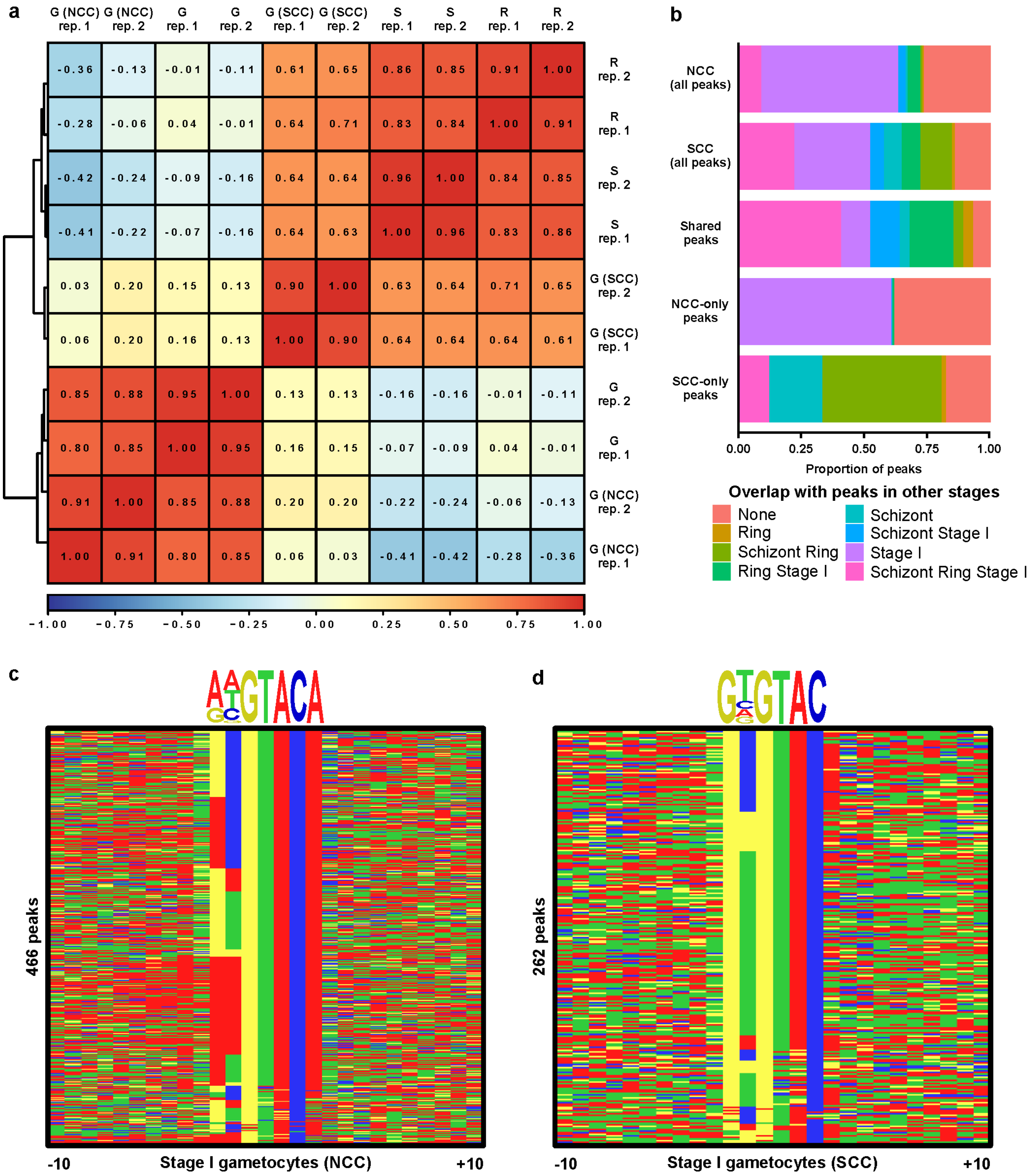

Supplementary Figure 4: Comparison of PfAP2-G occupancy in stage I gametocytes derived via NCC and SCC. a, Correlation plot for all PfAP2-G ChIP-seq experiments shown in Figures 1 and 2. Pearson correlation coefficients are shown for log2-transformed PfAP2-G ChIP/Input bigwig files. **b**, The proportion of all peaks in NCC stage I gametocytes, all peaks in SCC stage I gametocytes, peaks in both NCC and SCC stage I gametocytes, peaks in both, peaks in only NCC stage I gametocytes, and peaks in only SCC stage I gametocytes that overlap with peaks in other stages. **c** and **d**, DNA motif heatmaps of the ChIP-seq peaks and the top enriched

motifs (determined by DREME) for PfAP2-G in NCC and SCC stage I gametocytes (E values are 3.2e-94 and 2.5e-49, respectively).

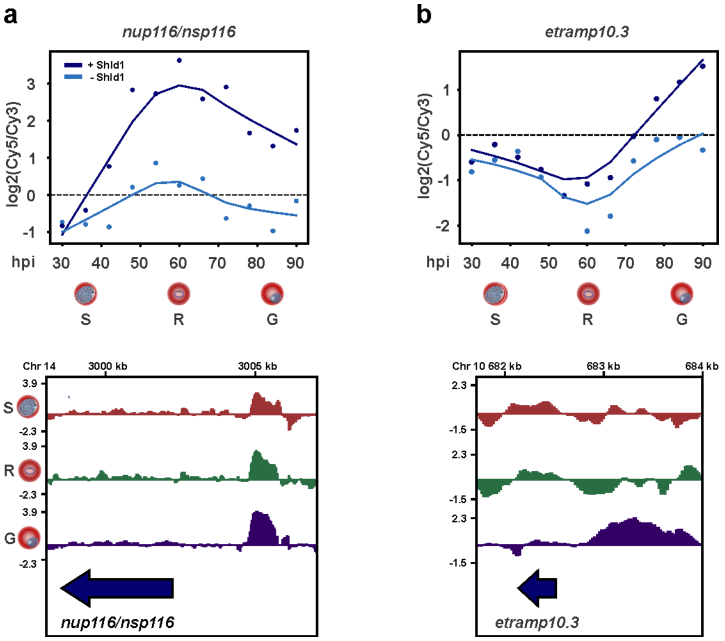

Supplementary Figure 5: Expression of two PfAP2-G target genes (*nup116/nsp116* and *etramp10.3*) and PfAP2-G binding over time. The top plots show expression of the gene of interest in AP2-G-DD +Shld1 (dark blue) and AP2-G-DD -Shld1 (light blue). The bottom panels show log2-transformed PfAP2-G ChIP/input ratio tracks for committed schizonts, sexual rings, and stage I gametocytes at the locus of interest. The blue arrow shows the coding sequence. ChIP-seq data are shown for one of the two biological replicates.

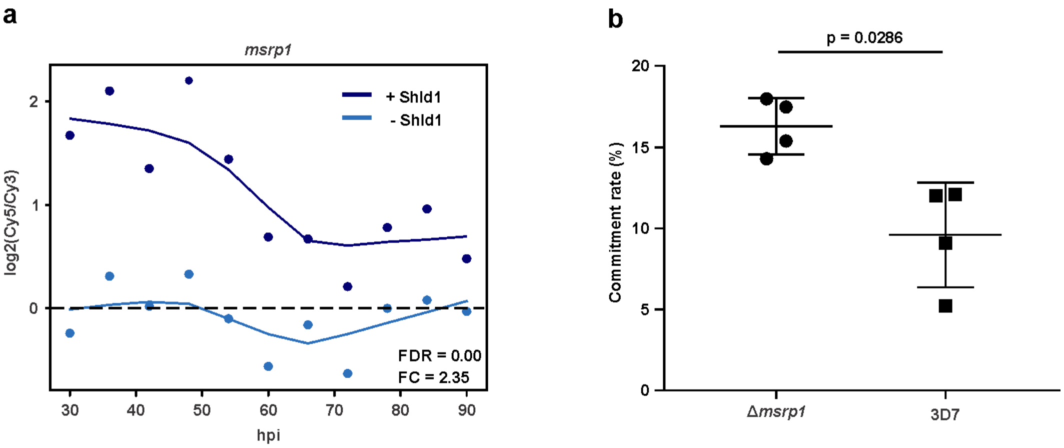

Supplementary Figure 6: **Expression of *msrp1* in committed cells. a**, Expression of *msrp1* in AP2-G-DD +Shld1 (dark blue) and AP2-G-DD -Shld1 (light blue). False discovery rate (FDR) determined using SAM and mean fold change (FC) across all time points are shown. **b**, The *msrp1* knockout line[3] has a higher commitment rate than its parent. The horizontal bars indicate the mean and standard deviation. n = 4 biologically independent samples. Statistical significance was tested for using the two-tailed Mann-Whitney U test. Source data are provided as a Source Data file.

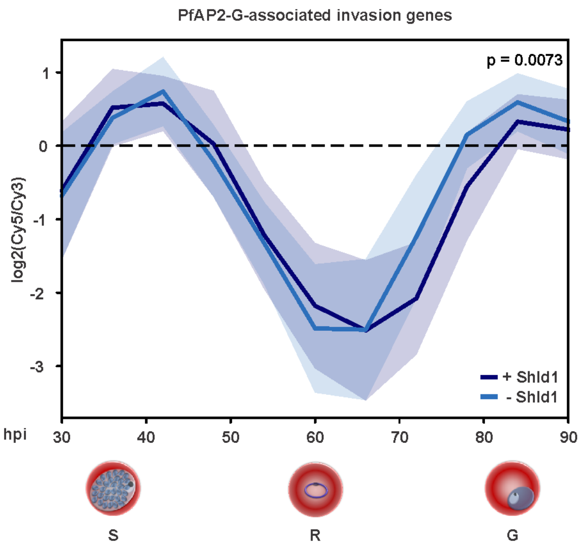

Supplementary Figure 7: Expression of invasion genes in AP2-G-DD +Shld1 and – Shld1. Plot shows mean temporal expression of the 36 invasion genes bound by PfAP2-G in AP2-G-DD +Shld1 (dark blue) and AP2-G-DD -Shld1 (light blue). The shaded regions indicate the standard deviation. Differences in expression between +Shld1 and -Shld1 were tested by two-way ANOVA. Source data are provided as a Source Data file.

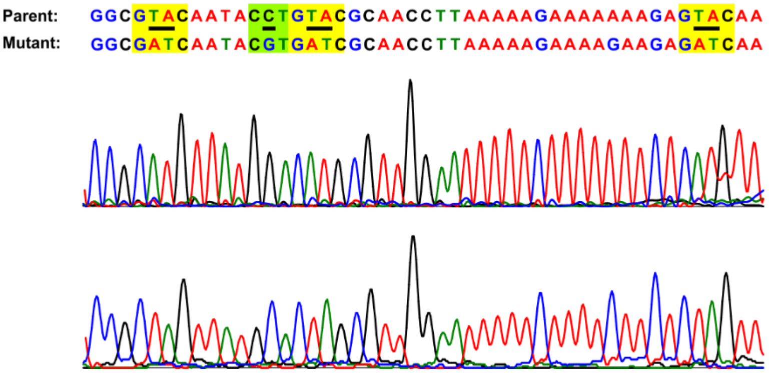

Supplementary Figure 8: DNA sequence chromatogram showing successful mutation of three of the eight PfAP2-G motifs upstream of *ap2-g*. The motifs are highlighted in yellow and the PAM is highlighted in green. Mutated nucleotides are underlined.

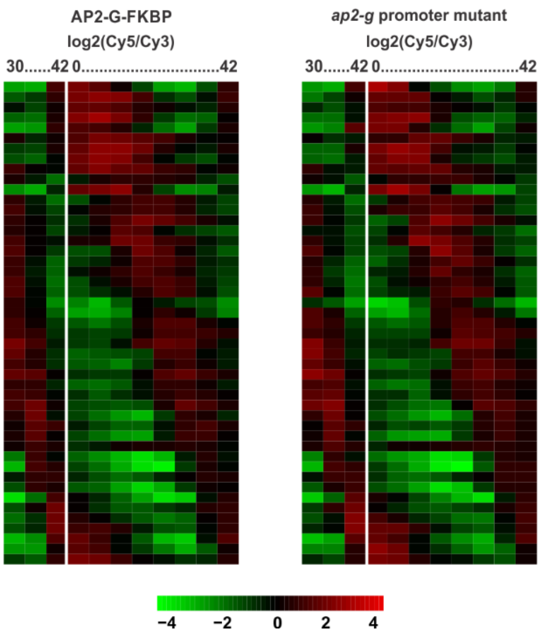

Supplementary Figure 9: Heatmaps showing expression of control genes[4] in AP2-G-FKBP and the *ap2-g* promoter mutant line. Both lines show a similar pattern of periodic gene expression, indicating that transcript levels are not globally affected and staging of the two lines is similar.

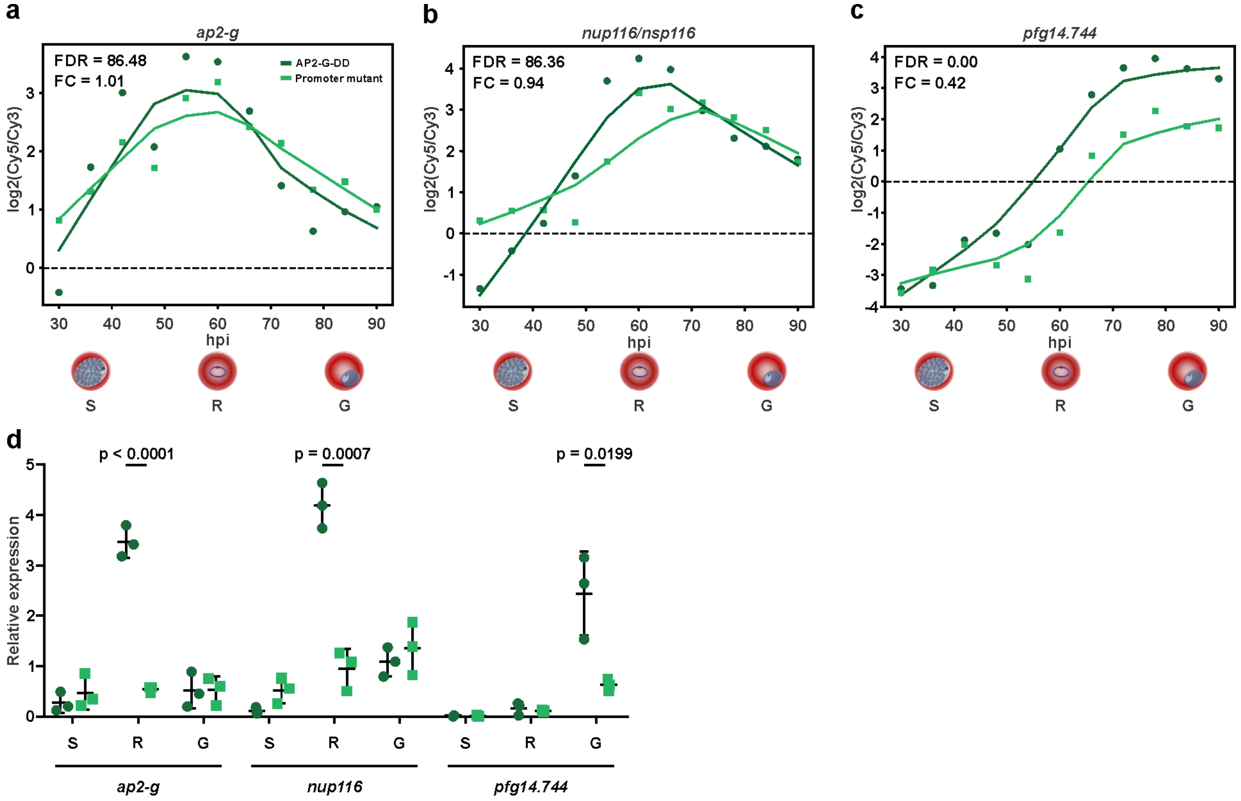

Supplementary Figure 10: Expression of PfAP2-G target genes in the *ap2-g* promoter mutant parasite line. Transcript levels of **a**, *ap2-g*, **b**, *nup116/nsp116*, and **c**, *pfg14.744* in the *ap2-g* promoter mutant parasite line (light green) and its parent AP2-G-DD (dark green) in DNA microarrays. False discovery rate (FDR) determined using SAM and mean fold change (FC) across all time points are shown. **d**, qRT-PCR analysis of RNA from three stages validates the DNA microarray data. The black bars indicate the mean and standard deviation. n = 3 biologically independent samples. Statistical significance was tested for using t-tests. Source data are provided as a Source Data file.

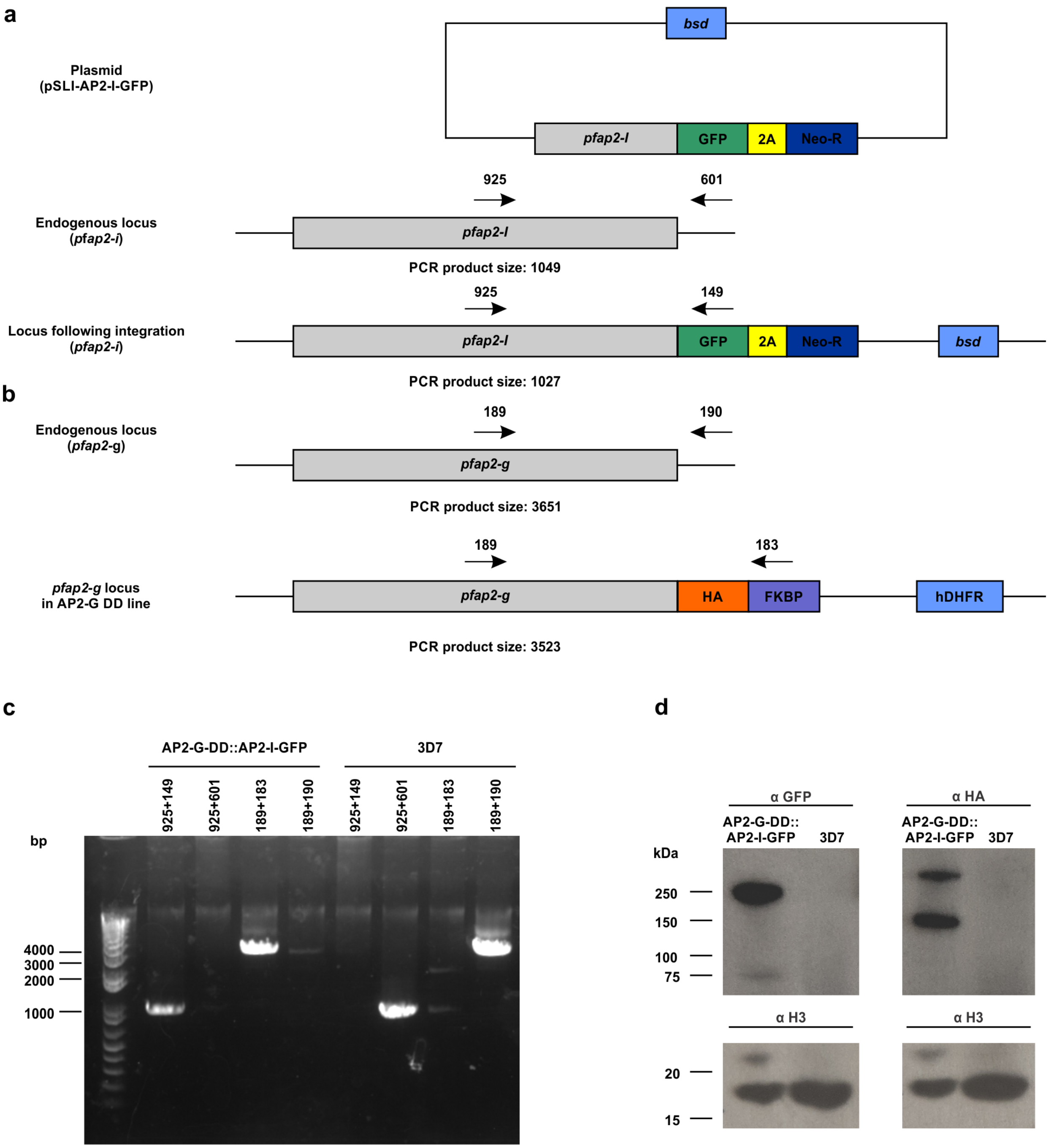

Supplementary Figure 11: Generation and validation of AP2-G-DD::AP2-I-GFP parasite line. **a**, The strategy used to tag PfAP2-I with GFP using selection-linked integration. Primers used to determine if integration had occurred are shown above the DNA sequences, and expected sizes of resulting PCR products are shown below. **b**, The *ap2-g* locus in wildtype and AP2-G-DD parasites. Primers used to confirm tagging of PfAP2-G are shown above the DNA sequences, and expected sizes of resulting PCR products are shown below. **c**, Genotyping PCR using the primers shown in a and b was performed on gDNA extracted from clonal AP2-G-DD::AP2-I-GFP parasites and 3D7. This confirmed that both genes had the intended integration. The uncropped original image is provided as a Source Data file. **d**, Western blot showing that the

AP2-G-DD::AP2-I-GFP line expresses both AP2-I-GFP (top left panel) and AP2-G-DD (top right panel), but 3D7 does not. H3 (bottom panels) was used as a loading control. The uncropped original image is provided as a Source Data file.

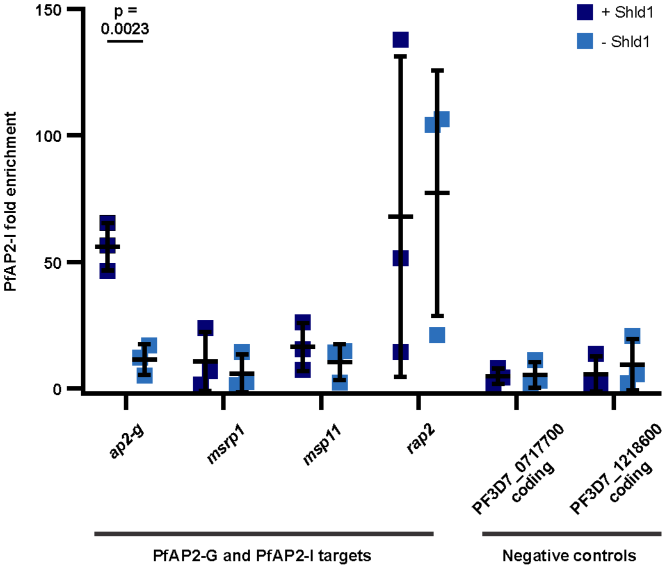

Supplementary Figure 12: PfAP2-I binding in the presence and absence of PfAP2-G. ChIP-qPCR performed in AP2-G-DD::AP2-I-GFP schizont-stage parasites shows that in the absence of Shld1 binding of PfAP2-I to some target gene promoters is reduced. Figure 5h shows the fold enrichment + Shld1 divided by fold enrichment - Shld1 for each replicate set. The black bars indicate the mean and standard deviation. n = 3 biologically independent samples. t-tests were performed to compare enrichment between +Shld1 and -Shld1.

Supplementary References

1 Kafsack, B. F. *et al.* A transcriptional switch underlies commitment to sexual development in malaria parasites. *Nature* **507**, 248-252, doi:10.1038/nature12920 (2014).

2 Bancells, C. *et al.* Revisiting the initial steps of sexual development in the malaria parasite Plasmodium falciparum. *Nature microbiology* **4**, 144-154, doi:10.1038/s41564-018-0291-7 (2019).

3 Kadekoppala, M., Ogun, S. A., Howell, S., Gunaratne, R. S. & Holder, A. A. Systematic genetic analysis of the Plasmodium falciparum MSP7-like family reveals differences in protein expression, location, and importance in asexual growth of the blood-stage parasite. *Eukaryotic cell* **9**, 1064-1074, doi:10.1128/ec.00048-10 (2010).

4 Kafsack, B. F., Painter, H. J. & Llinas, M. New Agilent platform DNA microarrays for transcriptome analysis of Plasmodium falciparum and Plasmodium berghei for the malaria research community. *Malaria journal* **11**, 187, doi:10.1186/1475-2875-11-187 (2012).

5 Poran, A. *et al.* Single-cell RNA sequencing reveals a signature of sexual commitment in malaria parasites. *Nature* **551**, 95-99, doi:10.1038/nature24280 (2017).

6 Kent, R. S. *et al.* Inducible developmental reprogramming redefines commitment to sexual development in the malaria parasite Plasmodium berghei. *Nature microbiology*, doi:10.1038/s41564-018-0223-6 (2018).

7 Lasonder, E. *et al.* Integrated transcriptomic and proteomic analyses of P. falciparum gametocytes: molecular insight into sex-specific processes and translational repression. *Nucleic acids research* **44**, 6087-6101, doi:10.1093/nar/gkw536 (2016).

8 Brancucci, N. M. B. *et al.* Probing Plasmodium falciparum sexual commitment at the single-cell level. *Wellcome open research* **3**, 70, doi:10.12688/wellcomeopenres.14645.4 (2018).
