# Supplementary material for: Dissecting the role of PfAP2-G in malaria gametocytogenesis
Source: Nat Commun. 2020 Mar 20;11:1503. doi: 10.1038/s41467-020-15026-0 (PMC7083873; doi:10.1038/s41467-020-15026-0)
Supplement: Supplementary file 3 — Reporting Summary [file 41467_2020_15026_MOESM3_ESM.pdf]

nature research

Corresponding author(s): Manuel Llinas

Last updated by author(s): Jan 25, 2020

Reporting Summary

Nature Research wishes to improve the reproducibility of the work that we publish. This form provides structure for consistency and transparency in reporting. For further information on Nature Research policies, see Authors & Referees and the Editorial Policy Checklist .

Statistics

For all statistical analyses, confirm that the following items are present in the figure legend, table legend, main text, or Methods section.

n/a	Confirmed	
☐	☒	The exact sample size (*n*) for each experimental group/condition, given as a discrete number and unit of measurement
☐	☒	A statement on whether measurements were taken from distinct samples or whether the same sample was measured repeatedly
☐	☒	The statistical test(s) used AND whether they are one- or two-sided<br>*Only common tests should be described solely by name; describe more complex techniques in the Methods section.*
☐	☒	A description of all covariates tested
☒	☐	A description of any assumptions or corrections, such as tests of normality and adjustment for multiple comparisons
☐	☒	A full description of the statistical parameters including central tendency (e.g. means) or other basic estimates (e.g. regression coefficient) AND variation (e.g. standard deviation) or associated estimates of uncertainty (e.g. confidence intervals)
☐	☒	For null hypothesis testing, the test statistic (e.g. *F*, *t*, *r*) with confidence intervals, effect sizes, degrees of freedom and *P* value noted<br>*Give P values as exact values whenever suitable.*
☒	☐	For Bayesian analysis, information on the choice of priors and Markov chain Monte Carlo settings
☒	☐	For hierarchical and complex designs, identification of the appropriate level for tests and full reporting of outcomes
☐	☐	Estimates of effect sizes (e.g. Cohen's *d*, Pearson's *r*), indicating how they were calculated

Our web collection on statistics for biologists contains articles on many of the points above.

Software and code

Policy information about availability of computer code

Data collection	qPCR data were collected using SDS v1.4 (Applied Biosytems). Normalized intensities from microarray data were obtained using Feature Extraction Software Version 9.5 (Agilent Technologies). Images from immunofluorescence assays were obtained using SlideBook 5.0 software (Intelligent Imaging Innovations).
Data analysis	GraphPad Prism 7 was used to generate plots showing commitment data and for statistical analysis. The Significance Analysis of Microarrays package in R was used for analysis of microarray data. For display of microarray data, heatmaps were generated using the R package pheatmap, scatterplots and lineplots were made using the Python package seaborn, and stacked bar plots where made using the R package ggplot2. ChIP-seq data were analysed using FastQC, Trimmomatic, BWA-MEM, samtools, closestbed (BEDtools), subtractbed (BEDtools), Multiple Intersect (BEDtools), MACS2, DREME, FIMO, and Tomtom. For display of ChIP-seq data, we used Integrative Genomics Viewer, plotProfile (deepTools), multiBigwigSummary (deepTools), plotCorrelation (deepTools), bamCompare (deepTools), enoLOGOS, and the cegr-tools program FourColorPlot (https://github.com/seqcode/cegr-tools).

For manuscripts utilizing custom algorithms or software that are central to the research but not yet described in published literature, software must be made available to editors/reviewers. We strongly encourage code deposition in a community repository (e.g. GitHub). See the Nature Research guidelines for submitting code & software for further information.

Data

Policy information about availability of data

All manuscripts must include a data availability statement. This statement should provide the following information, where applicable:

- Accession codes, unique identifiers, or web links for publicly available datasets
- A list of figures that have associated raw data
- A description of any restrictions on data availability

ChIP-seq data are deposited in the NCBI Sequence Read Archive (SRA) under the accession numbers GSE120448 [https://www.ncbi.nlm.nih.gov/geo/query/acc.cgi?acc=GSE120448] (PfAP2-G enrichment in committed schizonts, sexual rings, and stage I gametocytes), GSE134268 [https://www.ncbi.nlm.nih.gov/geo/query/acc.cgi?acc=GSE134268] (PfAP2-G enrichment in NCC and SCC stage I gametocytes), and GSE120488 [https://www.ncbi.nlm.nih.gov/geo/query/acc.cgi?

acc=GSE120488] (PfAP2-G and PfAP2-I enrichment in schizonts). Microarray data are deposited in the NCBI Gene Expression Omnibus (GEO) under the accession numbers GSE120990 [https://www.ncbi.nlm.nih.gov/geo/query/acc.cgi?acc=GSE120990] (AP2-G-DD + versus - Shld1) and GSE121312 [https://www.ncbi.nlm.nih.gov/geo/query/acc.cgi?acc=GSE121312] (AP2-G-DDap2-g mut and AP2-G-DD).

Field-specific reporting

Please select the one below that is the best fit for your research. If you are not sure, read the appropriate sections before making your selection.

☒ Life sciences ☐ Behavioural & social sciences ☐ Ecological, evolutionary & environmental sciences

For a reference copy of the document with all sections, see nature.com/documents/nr-reporting-summary-flat.pdf

Life sciences study design

All studies must disclose on these points even when the disclosure is negative.

Sample size	Two independent biological replicates were performed for each ChIP-seq experiment, in accordance with current ENCODE ChIP-seq guidelines. Microarray timecourse experiments were performed once due to the number of timepoints and samples being analysed limiting the feasibility of repeating these experiments. RT-qPCR was used to validate some of the microarray results. ChIP-qPCR and RT-qPCR experiments use three independent biological replicates. Commitment assays were performed using four independent biological replicates. The number of replicates used was based on the variability previously observed between replicates in similar experiments.
Data exclusions	For the microarray analysis, spots with red or green signal well below background were filtered out. Genes with data for less than 80% of timepoints were filtered out.
Replication	All reported findings could be reproduced in independent experiments (with the exception of the microarray timecourse experiments which were performed only once). The number of biological replicates used in each experiment is indicated in the figure legends and data for each individual replicate is shown. Though the microarray timecourse experiments were performed only once, qRT-PCR was performed to validate some results.
Randomization	N/A. Randomization was no suitable for any of the experiments performed.
Blinding	For commitment assays, counting of Giemsa smears was blinded. Blinding was not suitable for any of the other experiments performed.

Reporting for specific materials, systems and methods

We require information from authors about some types of materials, experimental systems and methods used in many studies. Here, indicate whether each material, system or method listed is relevant to your study. If you are not sure if a list item applies to your research, read the appropriate section before selecting a response.

Materials & experimental systems

n/a	Involved in the study
☐	☒ Antibodies
☐	☒ Eukaryotic cell lines
☒	☐ Palaeontology
☒	☐ Animals and other organisms
☒	☐ Human research participants
☒	☐ Clinical data

Methods

n/a	Involved in the study
☐	☒ ChIP-seq
☒	☐ Flow cytometry
☒	☐ MRI-based neuroimaging

Antibodies

Antibodies used

- rat monoclonal anti HA (Roche 11867431001, clone 3F10, multiple lot numbers including 27573500) was used for ChIP, Western blots, and IFAs
- rabbit polyclonal anti GFP (Abcam ab290, multiple lot numbers including GR3196305-1) was used for ChIP and Western blots
- rat IgG (Abcam ab37361, lot GR212762-1) was used for ChIP (negative control)
- rabbit IgG (Abcam ab46540, lot GR167963-1) was used for ChIP (negative control)
- AlexaFluor 546 coupled goat α-rat (ThermoFisher Scientific A11081, lot 584958) was used for IFAs
- AlexaFluor 488 coupled goat α-rabbit (ThermoFisher Scientific A11034, lot 1737902) was used for IFAs
- mouse α H3 (Abcam ab10799, lot number not available) was used for Western blots
- rabbit α aldolase HRP (Abcam ab38905, lot GR3242031-1 was used for Western blots
- goat anti-rat HRP conjugate (Abcam ab7097, lot GR311564-2) was used for Western blots
- goat anti-rabbit HRP conjugate (Millipore 12-348, lot DAM1698734) was used for Western blots
- goat anti-mouse HRP conjugate (Millipore 12349, lot number not available) was used for Western blots

Validation	All antibodies used in this study are commercially available. In a Western blot (Supplementary Fig. 8d) with anti HA and anti GFP, no bands were seen in wildtype parasite lysate and only bands corresponding to proteins of the expected size were detected in AP2-G-DD::AP2-I-GFP parasite lysate. For IFAs, secondary antibody only controls were performed and did not have any detectable fluorescent signal. ChIPs performed with rat and rabbit IgG (serving as negative controls) consistently had very low enrichment in the loci tested compared to the GFP and HA ChIPs.

Eukaryotic cell lines

Policy information about cell lines

Cell line source(s)	Previously published lines:<br>- AP2-G-DD was previously generated by our laboratory.<br>- 3D7Δmsrp1 and its parent 3D7 (used for commitment assays) were obtained from Tony Holder, the Francis Crick Institute.<br>- 3D7 (used as a control in Western blot) was obtained from MR4.<br><br>Lines generated in this study:<br>- AP2-G-DD-ap2-g mut: Generated in our laboratory as described in the Methods.<br>- AP2-G-DD::AP2-I-GFP: Generated in our laboratory as described in the Method
Authentication	Genotyping PCR and Sanger sequencing (if appropriate) was used to confirm the identify of transgenic cell lines, as described in the Methods.
Mycoplasma contamination	Cell lines were not tested for Mycoplasma contamination.
Commonly misidentified lines<br>(See ICLAC register)	N/A. None of the lines used are in the ICLAC Register of Misidentified Cell Lines.

ChIP-seq

Data deposition

- [x] Confirm that both raw and final processed data have been deposited in a public database such as GEO.
- [x] Confirm that you have deposited or provided access to graph files (e.g. BED files) for the called peaks.

Data access links<br>*May remain private before publication.*	PfAP2-G enrichment in committed schizonts, sexual rings, and stage I gametocytes: GSE120448<br>PfAP2-G enrichment in NCC and SCC stage I gametocytes: GSE134268<br>PfAP2-G and PfAP2-I enrichment in schizonts: GSE120488
Files in database submission	GSM3400933_AP2-G_S_HA_rep1.bigwig<br>GSM3400934_AP2-G_S_In_rep1.bigwig<br>GSM3400935_AP2-G_S_HA_rep2.bigwig<br>GSM3400936_AP2-G_S_In_rep2.bigwig<br>GSM3400937_AP2-G_R_HA_rep1.bigwig<br>GSM3400938_AP2-G_R_In_rep1.bigwig<br>GSM3400939_AP2-G_R_HA_rep2.bigwig<br>GSM3400940_AP2-G_R_In_rep2.bigwig<br>GSM3400941_AP2-G_G_HA_rep1.bigwig<br>GSM3400942_AP2-G_G_In_rep1.bigwig<br>GSM3400943_AP2-G_G_HA_rep2.bigwig<br>GSM3400944_AP2-G_G_In_rep2.bigwig<br>GSM3401470_GFP_rep1.bigwig<br>GSM3401471_GFP_rep2.bigwig<br>GSM3401472_HA_rep1.bigwig<br>GSM3401473_HA_rep2.bigwig<br>GSM3401474_Input_rep1.bigwig<br>GSM3401475_Input_rep2.bigwig<br>GSM3940782_NCC_HA_rep1.bigwig<br>GSM3940783_NCC_In_rep1.bigwig<br>GSM3940784_NCC_HA_rep2.bigwig<br>GSM3940785_NCC_In_rep2.bigwig<br>GSM3940786_SCC_HA_rep1.bigwig<br>GSM3940787_SCC_In_rep1.bigwig<br>GSM3940788_SCC_HA_rep2.bigwig<br>GSM3940789_SCC_In_rep2.bigwig<br>GSM3400933_AP2-G_S_rep1.bed.gz<br>GSM3400935_AP2-G_S_rep2.bed.gz<br>GSM3400937_AP2-G_R_rep1.bed.gz<br>GSM3400939_AP2-G_R_rep2.bed.gz

	GSM3400941_AP2-G_G_rep1.bed.gz<br>GSM3400943_AP2-G_G_rep2.bed.gz<br>GSM3401470_AP2-I_rep1.bed.gz<br>GSM3401471_AP2-I_rep2.bed.gz<br>GSM3401472_AP2-G_rep1.bed.gz<br>GSM3401473_AP2-G_rep2.bed.gz<br>GSM3940782_NCC_peaks_rep1.bed.gz<br>GSM3940784_NCC_peaks_rep2.bed.gz<br>GSM3940786_SCC_peaks_rep1.bed.gz<br>GSM3940788_SCC_peaks_rep2.bed.gz
Genome browser session (e.g. UCSC)	No longer applicable

Methodology

Replicates	All experiments were performed with two biological replicates.
Sequencing depth	For each sample, 8.5-20 million 150 bp single-end reads were generated. The numbers of reads generated for each sample are listed in Supplementary Table 1.
Antibodies	- rat monoclonal anti HA: Roche 11867431001, clone 3F10 (multiple lot numbers including 27573500)<br>- rabbit polyclonal anti GFP: Abcam ab290 (multiple lot numbers including GR3196305-1)
Peak calling parameters	macs2 callpeak MACS2 -t [IP file] -c [input file] -format=BAM --gsize 20000000 --bw 250 --mfold 5 50 --bdg --qvalue 0.01 --nomodel --extsize 200
Data quality	All reported peaks have a q-value <= 0.01. No peaks have a fold enrichment > 5. For each replicate, the known DNA motif bound by the transcription factor of interest was highly enriched, confirming the success of the ChIP. In addition, the fraction of reads in peaks (FRiP) was at least 1% for each replicate and thus the data meet ENCODE ChIP-seq guidelines.
Software	ChIP-seq data were analysed using FastQC, Trimmomatic, BWA-MEM, samtools, closestbed (BEDtools), subtractbed (BEDtools), MACS2, DREME, FIMO, and Tomtom. For display of ChIP-seq data, we used Integrative Genomics Viewer, plotProfile (deepTools), bamCompare (deepTools), multiBigwigSummary (deepTools), plotCorrelation (deepTools), enoLOGOS, and the cegr-tools program FourColorPlot (https://github.com/seqcode/cegr-tools).
