# Supplementary material for: Dissecting the role of PfAP2-G in malaria gametocytogenesis
Source: Nat Commun. 2020 Mar 20;11:1503. doi: 10.1038/s41467-020-15026-0 (PMC7083873; doi:10.1038/s41467-020-15026-0)
Supplement: Supplementary file 4 — Description of Additional Supplementary Files [file 41467_2020_15026_MOESM4_ESM.pdf]

Description of Additional Supplementary Files

Supplementary Data 1: Statistics for all ChIP-seq data generated in this study. For each sample, the total number of reads, the number of filtered and mapped reads, the percentage of filtered and mapped reads, coverage, the number of peaks called using MACS2, and the fraction of reads in peaks (FRiP) are listed.

Supplementary Data 2: PfAP2-G ChIP-seq data for AP2-G-DD schizonts. Table contains peaks identified in both biological replicates (worksheet 1) and each of the individual replicates (worksheets 2 and 3). In each case, the coordinates of each peak, the gene to its left, the gene to its right, the gene it is within (if any), and the peak location with reference to the adjacent genes (upstream of two genes, upstream of one gene, downstream of two genes, or within gene) are listed.

Supplementary Data 3: PfAP2-G ChIP-seq data for AP2-G-DD sexual rings. Table contains peaks identified in both biological replicates (worksheet 1) and each of the individual replicates (worksheets 2 and 3). In each case, the coordinates of each peak, the gene to its left, the gene to its right, the gene it is within (if any), and the peak location with reference to the adjacent genes (upstream of two genes, upstream of one gene, downstream of two genes, or within gene) are listed.

Supplementary Data 4: PfAP2-G ChIP-seq data for AP2-G-DD stage I gametocytes. Table contains peaks identified in both biological replicates (worksheet 1) and each of the individual replicates (worksheets 2 and 3). In each case, the coordinates of each peak, the gene to its left, the gene to its right, the gene it is within (if any), and the peak location with reference to the adjacent genes (upstream of two genes, upstream of one gene, downstream of two genes, or within gene) are listed.

Supplementary Data 5: Comparison of identified PfAP2-G binding sites with other studies identifying PfAP2-G targets using transcriptomic approaches. Comparisons with microarray data from PfAP2-G knockout lines (genes > 2-fold downregulated in both F12 and Δ*pfap2-g*)[1] (worksheet 1), scRNA-seq data from the AP2-G-DD line (all genes that are differentially expressed in PfAP2-G+ versus PfAP2-G- AP2-G-DD cells clusters 1-11)[5] (worksheet 2), RNA-seq data from a line over-expressing PbAP2-G[6] (all genes that are significantly upregulated at 6 hpi that have identified *P. falciparum* orthologues) (worksheet 3), and RNA-seq and proteomics data from male and female gametocytes (worksheet 4)[7].

Supplementary Data 6: PfAP2-G ChIP-seq data for AP2-G-DD stage I gametocytes derived via next-cycle conversion (NCC) and same-cycle conversion (SCC). Table contains peaks identified in both biological replicates for NCC (worksheet 1) and each of the individual replicates for NCC (worksheets 2 and 3), both biological replicates for SCC (worksheet 4) and each of the individual replicates for SCC (worksheets 5 and 6). In each case, the coordinates of each peak, the gene to its left, the gene to its right, the gene it is within (if any), and the peak location with reference to the adjacent genes (upstream of two genes, upstream of one gene, downstream of two genes, or within gene) are listed.

Supplementary Data 7: Shared and unique PfAP2-G binding sites for stage I gametocytes derived via next-cycle conversion (NCC) and same-cycle conversion (SCC). Table contains peaks identified in all four ChIPs (worksheet 1), in both NCC biological replicates but neither

SCC replicate (worksheet 2), in both SCC biological replicates but neither NCC replicate (worksheet 3), GO analysis of peaks found in NCC but not SCC (worksheet 4), and GO analysis of peaks found in SCC but not NCC (worksheet 5). Worksheets 2 and 3 also show whether or not the NCC- and SCC-unique peaks overlap with those found in other stages.

Supplementary Data 8: DNA microarray analysis of RNA from the AP2-G-DD line grown in the presence and absence of Shld1. Log2(Cy5/Cy3) ratios for each transcript at each timepoint for both conditions are listed. Significance analysis of microarrays (SAM) was used to identify differentially-expressed transcripts. Fold change in transcript levels (AP2-G-DD +Shld1 versus -Shld1) and false discovery rates are shown for timepoints 1-3, 4-7, 8-11, and 1-11.

Supplementary Data 9: Invasion genes identified in ChIP-seq and scRNA-seq studies. All invasion genes are listed by accession number and name (columns A and B) and comparisons with relevant datasets are in columns C-I. Column C: PfAP2-G schizont ChIP-seq in the AP2-G-DD::AP2-I-GFP line, column D: PfAP2-I schizont ChIP-seq in the AP2-G-DD::AP2-I-GFP line, column E: PfAP2-G schizont ChIP-seq in the AP2-G-DD line, column F: PfAP2-G ring ChIP-seq in the AP2-G-DD line, column G: PfAP2-G stage I gametocyte ChIP-seq in the AP2-G-DD line, column H: PfAP2-G NCC stage I gametocyte ChIP-seq in the AP2-G-DD line, column I: PfAP2-G SCC stage I gametocyte ChIP-seq in the AP2-G-DD line, column J: scRNA-seq up-regulated genes in AP2-G-DD and NF54 committed schizonts[5], and column K: scRNA-seq up-regulated genes in LysoPC-depleted Pf2004/164tdTom schizonts[8].

Supplementary Data 10: DNA microarray analysis of RNA from the *ap2-g* promoter mutant line and its parent (AP2-G-DD) grown in the presence of Shld1. Log2(Cy5/Cy3) ratios for each transcript at each timepoint for both lines are listed. Significance analysis of microarrays (SAM) was used to identify differentially-expressed transcripts. Fold change in transcript levels (AP2-G-DD$^{ap2\text{-}g\ mut}$ + Shld1 versus AP2-G-DD + Shld1) and false discovery rates are shown for timepoints 1-3, 4-7, 8-11, and 1-11.

Supplementary Data 11: PfAP2-G and PfAP2-I ChIP-seq data for AP2-G-DD::AP2-I-GFP schizonts. Table contains peaks identified in both biological replicates for PfAP2-G (worksheet 1), each of the individual replicates for PfAP2-G (worksheets 2 and 3), both biological replicates for PfAP2-I (worksheet 4), and each of the individual replicates for PfAP2-I (worksheets 5 and 6). In each case, the coordinates of each peak, the gene to its left, the gene to its right, the gene it is within (if any), and the peak location with reference to the adjacent genes (upstream of two genes, upstream of one gene, downstream of two genes, or within gene) are listed.

Supplementary Data 12: Shared and unique PfAP2-G and PfAP2-I binding sites. Table contains peaks identified in all four ChIPs (worksheet 1), in both PfAP2-G biological replicates but neither PfAP2-I replicate (worksheet 2), and in both PfAP2-G biological replicates but neither PfAP2-I replicate (worksheet 3).

Supplementary Data 13: Oligonucleotides used in this study.
